# Body weight at 10 years of age and change in body composition between 8 and 10 years of age were related to survival in a longitudinal study of 39 Labrador retriever dogs

**DOI:** 10.1186/s13028-019-0477-x

**Published:** 2019-09-09

**Authors:** Johanna Christina Penell, David Mark Morgan, Penny Watson, Stuart Carmichael, Vicki Jean Adams

**Affiliations:** 1 Department of Clinical Sciences, Faculty of Veterinary Medicine and Animal Science, Swedish University of Agricultural Sciences, 75007 Uppsala, Sweden; 2 EUKANUBA® UK, Spectrum Brands, Enterprise House, 42-45 Station Approach, West Byfleet, Surrey, KT14 6NE UK; 3 Present Address: Companion Animal Nutrition Consultant, Chemin de Sur-Beauvent 37, 1233 Bernex, Switzerland; 4 Department of Veterinary Medicine, University of Cambridge, Cambridge, CB3 0ES UK; 5 School of Veterinary Medicine, College of Medical, Veterinary and Life Sciences, University of Glasgow, 464 Bearsden Road, Glasgow, G61 1QH UK; 6 Vet Epi, Abbey Farm Cottage, Ixworth, Suffolk, IP31 2JP UK

**Keywords:** Cohort, Dogs, Cox, DEXA, Fat mass, Lean mass, Lean to fat ratio, Longevity, Healthspan, Sarcopenia

## Abstract

**Background:**

Overweight and obesity have been adversely associated with longevity in dogs but there is scarce knowledge on the relation between body composition and lifespan. We aimed to investigate the effects of body composition, and within-dog changes over time, on survival in adult Labradors using a prospective cohort study design. The dogs had a median age of 6.5 years at study start and were kept in similar housing and management conditions throughout. The effects of the various predictors, including the effect of individual monthly-recorded change in body weight as a time varying covariate, were evaluated using survival analysis.

**Results:**

All dogs were followed to end-of-life; median age at end-of-life was 14.0 years. Body composition was measured annually with dual-energy x-ray absorptiometer (DEXA) scans between 6.2 and 17.0  years. All 39 dogs had DEXA recorded at 8, 9 and 10 years of age. During the study the mean (± SD) percent of fat (PF) and lean mass (PL) was 32.8 (± 5.6) and 64.2 (± 5.5) %, respectively, with a mean lean:fat ratio (LFR) of 2.1 (± 0.6); body weight (BW) varied from 17.5 to 44.0 kg with a mean BW change of 9.9 kg (± 3.0). There was increased hazard of dying for every kg increase in BW at 10 years of age; for each additional kg of BW at 10 years, dogs had a 19% higher hazard (HR = 1.19, P = 0.004). For the change in both lean mass (LM) and LFR variables, it was protective to have a higher lean and/or lower fat mass (FM) at 10 years of age compared to 8 years of age, although the HR for change in LM was very close to 1.0. For age at study start, older dogs had an increased hazard. There was no observed effect for the potential confounders sex, coat colour and height at shoulders, or of the time-varying covariate.

**Conclusions:**

These results suggest that even rather late-life control efforts on body weight and the relationship between lean and fat mass may influence survival in dogs. Such “windows of opportunity” can be used to develop healthcare strategies that would help promote an increased healthspan in dogs.

## Background

Most of us recognise that the longevity of our companion dogs has increased. Figures reported from a 25 years period in the United Kingdom (UK) showed that the average age of dogs had increased from 4.8 to 6.0 years, an increase of 25%; there was also an increase in the proportion of dogs living beyond 8 years from 24 to 34% [1]. This improvement in longevity can be explained by several factors, including advances in veterinary medicine, improvements in nutritional support and owner interest in seeking better veterinary and husbandry care. For most owners the thought that their dog could achieve an exceptional age, whilst still having a satisfactory quality of life, is an attractive prospect. In order to maximise both the quality and quantity of life experienced, an ultimate aim should be to extend both a dog’s lifespan and healthspan, the latter being defined as the period of life during which an individual is generally healthy and free from serious disease [2].

Dogs provide valuable companionship and contribute to enhanced life quality for their owners. In addition, dogs provide a model of ageing for humans [3] as they share a similar environment, are exposed to the same pollutants, receive comparable medical care [3, 4], age faster [3] and their ageing is also similar in many ways to human ageing [5, 6]. For example, research has been undertaken to identify key cellular and biomolecular dysfunctions that drive inflammageing and muscle wasting disorders in humans [7] as well as investigating inflammageing, and its intervention, in dogs [8, 9]. Inflammageing is a term used to describe the low-grade, chronic, systemic inflammation, in the absence of overt infection (“sterile” inflammation), that occurs with ageing [10]. The translational aspect of canine ageing as a model for human ageing is further captured by the concept of One Health, a worldwide strategy for expanding interdisciplinary collaborations and communications in all aspects of health care for humans, animals and the environment. Although specific diseases, such as Alzheimer’s [11], and other factors related to ageing, such as nutritional strategies, have been reported in dogs [12], it is not yet clear what other factors may be related to increased longevity.

It has been known for many years, that one of the most effective strategies for increasing healthspan across numerous species is through calorie restriction [3, 13, 14]. There are several publications that report on other beneficial effects of calorie restriction in dogs; for example, a major effect of lifetime calorie restriction on the development and progression of osteoarthritis (OA) in both hip and elbow joints of Labrador retrievers has been shown [15–17]: this seminal study was carried out in the USA from 1987 to 2001 and involved a birth cohort of 48 Labrador puppies randomly assigned to a feeding group at 8 weeks of age and followed until end-of-life. The ‘control’ group (n = 24) was initially fed ad libitum and then fed a reduced amount of food from 3.25 years of age onwards to prevent excessive weight gain and based on calculations of energy requirements to maintain an ideal body weight for a large breed dog, whilst the ‘restricted’ group (n = 24) was still fed 25% less than their sex and body weight (BW) matched sibling pair. A recent publication [18] reported and compared median survival times (MST) of four groups of Labradors that included the two aforementioned groups, a group of 542 privately owned UK dogs that died after 3.5 years of age, and as reported herein, a cohort study group that followed 39 dogs from mid-adulthood to end-of-life and fed with an aim to maintain a body condition score (BCS) between 2 and 4 on a 5-point scale [19]. The cohort of 39 dogs had an MST of 14.0 years which was significantly longer than the MSTs for the other three groups. The birth cohort experienced an MST of 11.1 years for the ‘control’ group which was significantly shorter than the MST of 12.9 years for the ‘restricted’ group. The final group, reported from the UK survey, showed an MST of 12.6 years which was not significantly different from the ‘restricted’ group. Other studies of owned Labradors have reported similar median ages of 12 years at end-of-life [20–22]. In spite of this, detailed knowledge on the long-term effect of calorie restriction on other aspects of healthy ageing, such as body composition, is still missing.

Whilst a long healthspan can be enhanced by reducing the frequency or delaying the onset of diseases such as cancer, it has been suggested that a major influence in increasing longevity will be to slow down the ageing process [3, 23, 24]. The ageing process that leads to a decline in function of most organs and tissues, including sarcopenia, cognitive decline, diabetes and osteoarthritis may be similar in dogs and humans [3]. Sarcopenia, the gradual and progressive loss of muscle mass and function in absence of disease, is a key process in ageing humans [25, 26] and dogs [27, 28]. Research has emphasised the importance for humans of maintaining a lean body condition by preserving muscle mass, and losing fat mass, to prevent development of lifestyle-related diseases and/or impaired mobility that negatively impacts a healthy life expectancy [29, 30]. In dogs, preliminary results on average changes in body composition up to 13 years of age have been described for the cohort of 39 Labradors, reported on earlier [18, 19], although this did not follow all dogs until end-of-life and the analysis was not adjusted for potential confounding variables [19]: dual-energy x-ray absorptiometer (DEXA) scans revealed that the increase in absolute (kg) fat mass (FM) was significantly lower for dogs surviving ≥ 13 to ≤ 15.5 years of age compared with dogs that died before they reached 13 years, whilst all dogs lost a similar but non-significant amount of absolute lean mass (LM). Dogs that lived longer than 13 years also had a significantly smaller change in both the percent body fat increase, and percent body lean decrease, than dogs living up to 13 years. We also showed that the rate of change in the fat to lean ratio was related to longevity where dogs living < 13 years had a larger change in fat mass compared to lean mass, than dogs living ≥ 13 years. Other studies have reported inconsistent associations between body composition variables at different ages and age at end-of-life [31, 32], including a moderate significant negative linear correlation (*r*^2^ = 0.41) between lean fat ratio (LFR) and age in 40 Labradors (age range 2.0 to 13.0 years) [32] although this was not corroborated in a similar study of 35 Labradors (age range 2.0 to 14.3 years) that showed a small positive correlation between FM and LM (*r*^2^ = 0.152) but no association of either FM or LM with age [31]. In the birth cohort group of 48 Labradors reported on earlier, annual DEXA scans performed near their date of birth from 6 years of age to just prior to the end-of-life scan showed a highly predictive effect (P < 0.002) of fat and lean composition with early end-of-life. A high percent fat mass (PF) predicted an early end-of-life 1 year before end-of-life whilst a high percent lean mass (PL) had a protective effect [33]. Unfortunately, the lack of a longitudinal study design (and use of repeated measures) in some of the previous studies prevented an evaluation of the effect of changes in body composition of individual dogs over their lifetime. Detailed knowledge of the impact on survival of changes in LM, FM and body weight mass (BW), the relationship between lean and fat across the lifespan of dogs and the effect of potential confounding variables is still lacking. The aim of the present longitudinal study was to investigate the effects of body composition and within-dog changes over time on survival in the previously described cohort of 39 adult Labrador retrievers using a repeated-measures prospective cohort design.

## Methods

### Study participants

The participating 39 neutered adult Labrador retrievers (12 males, 27 females) were all part of an original study designed as a clinical trial to test a novel energy restriction mimetic in the form of a dietary supplement, mannoheptulose. Details of the original study protocol have been previously described [19]; specific details are included to allow understanding of the objectives of the present study. All dogs were acquired from private breeders or one United States Department of Agriculture-inspected provider. One breeder provided 32 (82%) of the dogs^1^ from the mating of a total of 12 sires and 19 dams. There were four pairs of littermates, and two groups of three littermates amongst the 39 dogs, all six of these groups came from this one main breeder. For the 32 dogs, from the single breeder, their husbandry (feeding and kennelling) and medical care (deworming and vaccinations) were kept consistent from birth and throughout early adulthood prior to them joining the study.

During the study all dogs were housed in indoor kennels kept within an agreed temperature, humidity and air flow range. Additionally, each kennel had access to a partially covered outdoor run. The dogs were provided with size-appropriate dog toys (for the indoor kennels) and equipment designed to provide environmental enrichment was provided in the outdoor exercise areas: e.g. agility course apparatus, wading pools, a variety of dog toys and shaded areas. Twenty minutes of individual daily socialisation of all dogs took place with a qualified animal welfare specialist. Social groups of three to six dogs were exercised daily for a further 30 min minimum in the large gravel lined or grass outdoor exercise area. Compatible groups of dogs had 24-h access to each other through their partially covered outdoor run that interconnected neighbouring kennels to allow for additional socialisation and exercise. Grooming was performed bi-weekly, bathing quarterly and each dog had access to regular veterinary care. The study design was approved by the Institutional Animal Care and Use Committee (IACUC) of Procter & Gamble (P&G) Pet Care (Mason, OH, USA) and the accommodation facility where the dogs were housed was accredited by the Association for Assessment and Accreditation of Laboratory Animal Care [19].

### Study preparation and feeding

The dogs underwent an acclimatisation period from May 2003 up to the start of the study on 16 July 2004. During this time the dogs were fed a nutritionally complete and balanced dry diet with 27.2% dry matter (DM) protein and 14.9% DM fat, see Adams et al. [19] for details. All dogs were surgically neutered in 2003 prior to the start of the study period. The mean and median age at neutering was 5.5 years (range 4.3–7.5 years) [19]; one male dog had been neutered at 4.4 years of age just prior to being recruited in May 2003. The dogs entered the study in 2004 at a median age of 6.5 years (± 0.9) and a mean BW of 28.7 kg (± 4.3 kg). During the study the aim was to maintain a body condition score (BCS) of 3 based on a 5-point scale, although a score of 2 or 4 was accepted to avoid oscillating changes in feeding amounts and body weight. During the study, BCS was assessed by trained staff on a quarterly basis to enable adjustment of daily food allowances to maintain a BCS between 2 and 4 [19]. The daily food allowance could also be changed by the supervising veterinarian for medical purposes. The daily ration of food was weighed for each dog, divided daily into two equal portions and offered in stainless steel food bowls. At the start of the study on 16 July 2004 all 39 dogs had been transitioned to a complete and balanced dry study diet that incorporated nutritional components found in EUKANUBA^®^ Senior Maintenance for large breed dogs^2^. The diet, which was fed during the entire study, had 26.7% DM protein, derived principally from animal based sources and 16.2% DM fat. The formula included low glycaemic index grains, l-carnitine, the prebiotic Fructo-oligosaccharide and polyphosphate technology for reducing tartar build-up. Dogs were separated for feeding and food intake was recorded daily. Fresh water was constantly available using automatic adjustable water bowls mounted on the side of each housing unit [19].

### Quality of life and end-of-life decisions

In addition to routine veterinary examinations, each dog was monitored daily by their animal welfare specialist and the animal husbandry staff; any health related concerns were brought to the attention of the attending healthcare staff (veterinarian and veterinary nurse). The healthcare staff would then initiate a quality of life (QoL) assessment. Assessments were conducted by animal welfare specialists, animal husbandry staff and healthcare staff (attending veterinarian, veterinary nurses) to assess quality of life using a 10-point Likert scale ratings to assess food/water intake (hunger/thirst), pain/discomfort, mobility, hygiene, happiness and number of “good days” [34]. If the QoL was in decline then a consultation would take place within a defined team that included the study director and a number of veterinarians, both from the pet care division and from the wider company^3^ who were not involved in the study. The team were blinded to the dog’s identity and only the on-site attending veterinarian knew the identity. Decisions about administration of any medications, and end-of-life decisions, were made by the group based on the QoL assessment and the dog’s overall well-being and if it was compromised. The study director and the on-site veterinarian and veterinary nurse who worked at the Animal Care Center (ACC) where the dogs were housed, and the QoL assessment protocol, remained constant throughout the study period. Two of three company veterinarians also remained constant over the course of the study. The end-of-life decision was made with the attending veterinarian, veterinary nurse, study director, and a minimum of one non-ACC veterinarian. The purpose of this approach was to maintain objectivity in making end-of-life decisions by balancing any personal bias from the attending veterinarian and/or veterinary nurse with the expertise from licensed veterinarians (not involved in the study) and the study director. The on-site attending veterinarian was allowed to make an immediate end-of-life decision if there was a rapid deterioration in any dog’s health.

### BW and body composition

BW was measured every 2 weeks and whole body composition measures were obtained during the acclimatisation period in 2003 and annually from 2005 to 2015 using a DEXA scanner (Model Delphi-A, Serial No. 70852; Bedford, MA). Dogs were fasted for a minimum of 12 h prior to being weighed and sedated for the DEXA scan using a pre-anaesthetic combination of acepromazine (PromAce^®^ Injectable, Fort Dodge, Fort Dodge, IA; 0.55 mg/kg intramuscular injection) and atropine sulfate (Med-Pharmex, Pomona, CA; 0.04 mg/kg subcutaneous injection). Dogs were then anaesthetised via a secured intravenous catheter to administer propofol (Propoflo^®^, Abbot Labs, Chicago, IL; 7 mg/kg). Dogs were then intubated with an endotracheal tube, to deliver 100% oxygen and routine anaesthetic monitoring was performed. Dogs were positioned in sternal recumbency with hind limbs extended caudally and a calibration was completed prior to each DEXA scan, after which measurements were taken using the whole body scanner (single-beam mode). Oxygen was continued for several minutes after the scan was completed before moving the dogs to a recovery cage; the endotracheal tube was removed once the swallowing reflex was regained.

### Data analysis

#### Outcome variable and risk factors

Labrador coat colour was black, yellow or chocolate, sex was either female or male and all were neutered prior to the study commencing in mid-July 2004. Age at neutering and age at start of study were recorded as well as the height at the shoulders in centimetres and as a categorical variable based on tertiles of the data. Whole body composition measures obtained from the DEXA scans included total body mass as the fasted BW just prior to the scan (kg), total FM (kg), total LM (kg), PF and PL. The LFR was estimated as LM divided by FM. For DEXA derived variables at specific ages, the time points used were 8, 10 and 12 years of age based on including observations ± 0.5 years from the exact age; however, for the 8-year time point, two observations of 7.4 years and two observations of 8.8 years were included to maintain a complete data set; for the 10-year time point, one observation of 9.1 years was included.

For continuous variables, mean [± 1 standard deviation (SD)] are presented as descriptive statistics. Dogs were previously classified into three groups derived from tertiles of lifespan data: ‘expected’ (n = 13) when they experienced a lifespan of ≥ 9 to ≤ 12.9 years, ‘long’ (n = 15) when they experienced a lifespan between ≥ 13 and ≤ 15.5 years and ‘exceptional’ (n = 11) when they achieved ≥ 15.6 years and beyond, as presented in a previous publication [19]. The value of 15.6 years to describe the start of an exceptional age for the Labradors was defined as the breed’s typical or average lifespan of 12 years, as determined by a consensus group of canine experts, plus 30% [19, 35]. Percent body fat against age and percent body lean against age are presented by longevity category in polynomial smooth plots for illustrative purposes (all dogs in the present analysis had reached end-of-life compared to our earlier DEXA analysis where 26 dogs in the long (n = 15) and exceptional (n = 11) groups were still alive [19]).

#### Statistical analyses

Kaplan–Meier survival curves were used to examine the effect of sex, coat colour and height at shoulder on survival times. Survival analysis using Cox proportional hazards regression was performed to estimate the effect of potential predictors on “time to end-of-life”. The variables age at study start, sex, coat colour, shoulder height, starting, maximum and minimum BW during the study, age at minimum BW during study, age at maximum BW during study, and the body composition variables (at specific ages of 8, 10 and 12 years and also as linear changes over 2-year intervals for 8–10 years, 10–12 years) were examined for potential influence on the age of end-of-life. The selection of the variables for potential inclusion in a full model included a two-stage process: (1) univariate Cox regression analysis selected variables with P < 0.25 followed by (2) correlation (Pearson’s correlation coefficient = *r*) analysis to select variables that did not show collinearity (*r* < 0.7). Time at risk was measured in days from study start in July 2004 until end-of-life. For the main model, variables with data available for all 39 dogs were examined for potential influence on overall survival. The final Cox regression model was selected based on step-wise reduction from the full model until only variables with P < 0.05 remained. All models were adjusted for clustering of dogs within litter. All two-way interactions of the main effects were tested.

#### Validation of the model

Model diagnostics were performed on the final model evaluating the assumption of proportional hazard using the test for proportional hazards based on the Schoenfeld residuals for each variable in the model, the goodness-of-fit test and by graphing the survival function for the mean of all covariates in the final model to illustrate the impact of an increased hazard due to a covariate of interest [36, 37]. Sensitivity analyses were performed in three ways: (i) excluding the observations that were outside the ± 0.5 year range for ages 8 (n = 4) and 10 (n = 1); (ii) including data at 12 years (± 0.5 years) into the model which reduced the number of dogs included to 37; and (iii) taking the monthly average BW change into account as a time-varying covariate. The level of significance for all statistical tests was set at P < 0.05. Statistical analysis was done using Stata SE v14.2 (Stata Corporation).

## Results

### Descriptive statistics

DEXA data included 12 calendar years (2003, 2005–2015) of which the eleven years within the study period (2005–2015) were included in this analysis. Age at DEXA scans within this study ranged from 6.2 to 17.0 years. All 39 dogs had DEXA recorded at 8, 9 and 10 years of age and 37 dogs also had a DEXA scan at 12 years (Table 1). One dog was found dead in its kennel and the other 38 dogs underwent euthanasia due to morbidities affecting their quality of life; the exact date of the end-of-life was used as the end point of the study (Table 2).

**Table 1:** Demographic and descriptive data for 39 dogs followed until end-of-life, including body composition data

Variable	Category	N	%
Sex	Male	12	31
	Female	27	69
Colour	Black	14	36
	Chocolate	3	8
	Yellow	22	56

Variable	N	Mean (± SD)	
Height at shoulders (cm)	37	55.0 (3.0)	
Age at study start (years)	39	6.8 (0.9)	
Age at neutering (years)	39	5.5 (0.8)	
Age at end-of-life (years)	39	14.2 (2.0)	
Body weight at study start (kg)	39	28.7 (4.3)	
Body weight at 8 years (kg)	39	30.4 (4.0)	
Body weight at 10 years (kg)	39	32.1 (4.6)	
Body weight at 12 years (kg)	37	31.5 (4.0)	
Body condition score at 8 years	20	3.3 (0.4)	
Body condition score at 10 years	39	3.4 (0.5)	
Body condition score at 12 years	37	3.4 (0.5)	
Total fat mass (%) (n_dogs_ = 39)	275	32.8 (5.6)	
Total fat mass (kg) (n_dogs_ = 39)	275	10.1 (2.7)	
Total lean mass (%) (n_dogs_ = 39)	275	64.2 (5.5)	
Total lean mass (kg)	275	19.5 (2.7)	
Change lean mass 8 to 10 years	39	− 392.4 (741)	
Ratio lean:fat (g)	275	2.1 (0.6)	
Change lean:fat 8 to 10 years	39	− 0.3 (0.4)

**Table 2:** Causes of end-of-life for 39 dogs

Cause of death	N	(%)	Median age at death, in years (min–max)	Comments
Cancer	16	(41%)	13.9 (9.7–17.9)	5 hemangiosarcoma, *2 hepatocellular carcinoma*, 1 mast cell tumour + *1 mast cell tumour*, 2 urinary tract cancer and 1 each of lymphosarcoma, osteosarcoma, plasma cell tumour, adenocarcinoma and prostatic cancer
Gastrointestinal disease	4	(10%)	13.5 (12.8–14.1)	1 each of mega-oesophagus, gastrointestinal (GI) inflammation, gastric dilatation volvulus and found dead in kennel [enteritis, colitis + protein-losing enteropathy (PLE)]
Heart, kidney, liver and/or pancreas disease	5	(13%)	15.2 (13.6–17.6)	2 chronic kidney disease, 1 myocardial fibrosis + *1 other heart disease* + *1 renal, GI, liver and pancreatic pathology*
Musculoskeletal	8	(20%)	13.8 (12.1–15.8)	7 osteoarthritis + 1 intervertebral disc disease
Other	3	(8%)	13.9 (11.7–15.2)	2 seizures + 1 septicaemia
Quality of life	3	(8%)	15.8 (14.8–17.0)	With no proximate cause identified on post-mortem examination

Cause of end-of-life for the last 5 dogs were not included in Adams et al. (ref. [19]) and have been added to this table in italics

**Fig. 1:**
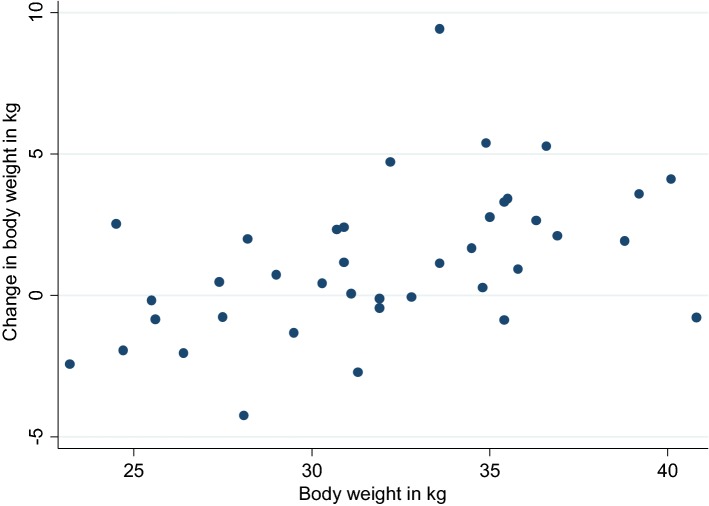
The relationship between body weight at 10 years and the body weight change between 8 and 10 years for 39 Labrador retrievers (Pearson’s correlation coefficient = 0.48, P = 0.02)

Body composition varied for all dogs up to their end-of-life. The mean minimum and maximum body weight during the study (irrespective of age or time of weight) were 24.8 kg (± 3.6) and 34.7 (± 3.9), respectively. The dog that was heaviest during the study reached a maximum BW of 44.0 kg approximately 1 year into the study at 6.8 years of age; he was also the tallest dog (61 cm shoulder height) and he died at 13.2 years of age. The mean individual BW change during the study was 9.9 kg (± 3.0). The dog that was lightest during the study reached the minimum BW of 17.5 kg at 14 years of age; she then slowly increased her body weight to 22 kg at her death at 17 years of age; she was one of the two smallest dogs (49.5 cm shoulder height). The body weight at 10 years and the body weight change between 8 and 10 years was moderately correlated (Pearson correlation r = 0.48) (Fig. 1) The mean individual minimum BW for all 39 dogs was similar to that for the 19 dogs that had > 10 kg BW change during the study, although these 19 dogs lived somewhat longer with a median age of 14.0 years at end-of-life; eight of these 19 dogs were in the exceptional group (lived > 15.5 years) and included three of the four dogs that lived > 17 years.

**Fig. 2:**
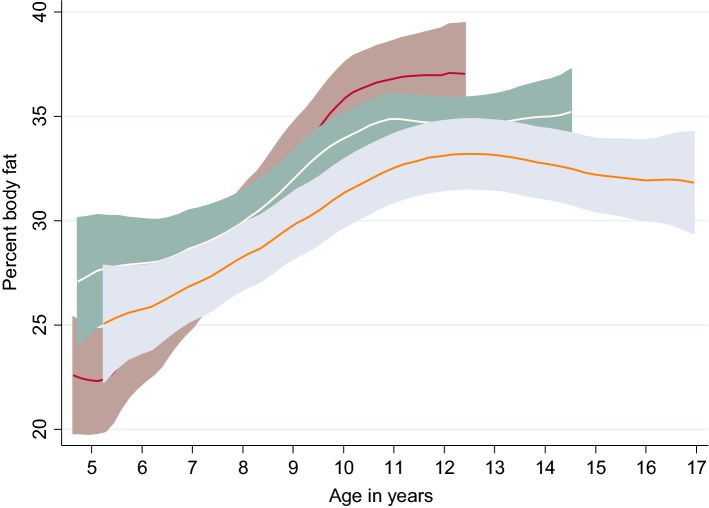
Polynomial smooth plot of average percent fat (95% CI) by age for each longevity category. Groups are based on tertiles of survival: expected (red line), long-lived (white line) and exceptional (orange line) for 39 dogs including all available DEXA data. Mean percent fat increased until approximately 10 years of age for dogs in the expected and 11 years for the long-lived groups and up to 12 years of age for exceptional group. After these ages, the percent fat stabilised

**Fig. 3:**
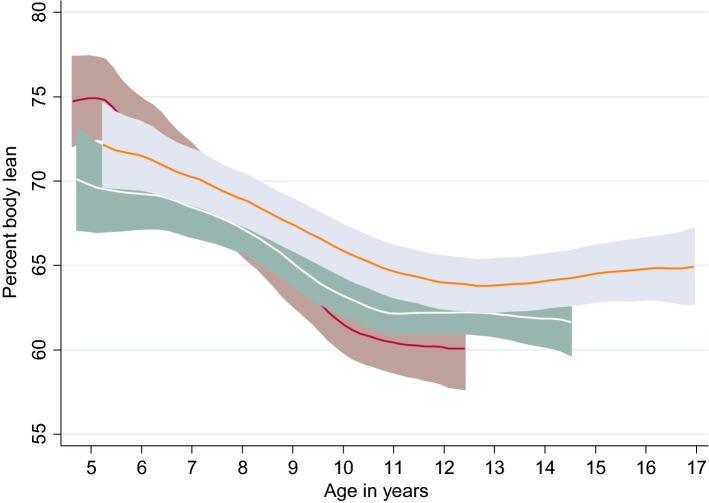
Polynomial smooth plot of average percent lean (95% CI) by age for each longevity category. Groups are based on tertiles of survival: expected (red line), long-lived (white line), and exceptional (orange line) for 39 dogs including all available DEXA data. The change in percent lean showed a similar but mirrored pattern to percent fat (Fig. 2) with a decrease in percent lean until approximately 11 years for the expected and long-lived groups and 12 years for the exceptional group, respectively. After this, the percent lean stabilised

**Fig. 4:**
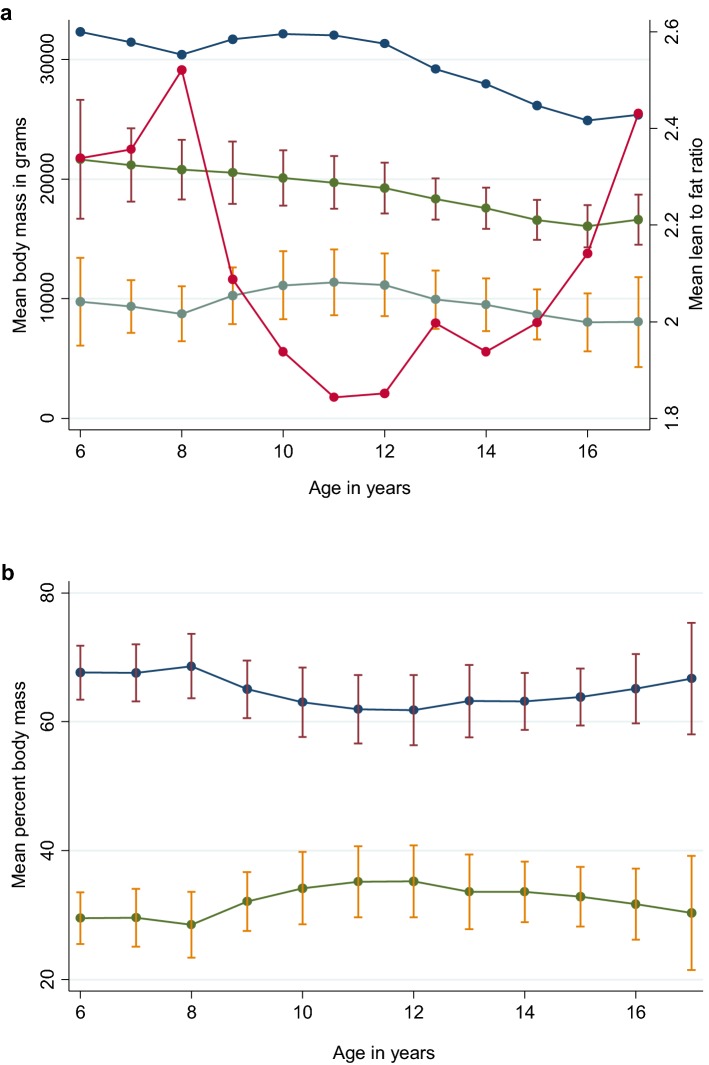
**a** Lean mass, fat mass, body weight and lean to fat ratio by age. The mean values and ± 1 standard deviation (bars) for lean mass (green line with dark red bars), and fat mass (grey line with orange bars), and mean body weight (blue line) as weight in grams on left y-axis and the lean to fat ratio (red line) on right y-axis, by age for 39 Labradors followed from a median age at the start of the study of 6.5 years until end-of-life. **b** Lean percent and fat percent by age. The mean values and ± 1 standard deviation (bars) for lean percent (red line with blue bars) and fat percent (orange line with green bars), by age for 39 Labradors age showed a similar pattern as lean and fat mass in **a**

The PF against age by longevity category is presented in Fig. 2. Between 7 and 10 years, expected dogs showed an average increase in PF of 4.2% per 1 year increase in age, whereas long and exceptional dogs showed average increases in PF of 3.2% and 1.6%, respectively, per year; the difference between the longevity groups was statistically significant when evaluated using a linear regression model (P = 0.02). The decrease in PL by age mirrored the gain in PF (Fig. 3). The mean values (± 1 SD) of LM and FM are shown with the LFR and BW by age (Fig. 4a). Only seven dogs had a positive change in LFR from 8 to 10 years of age. Mean fasted BW values, measured just prior to each DEXA scan, followed the same pattern as fat mass changes by age but were ~ 22 kg higher at each age until ~ 12 years age, after which the difference was smaller. The pattern of mean values (± 1 SD) of PL and PF (Fig. 4b) were similar to those for LM and FM.

### Survival analysis

In total, the 39 dogs contributed 105,636 days at risk during the study, with a median of 2715 days at risk and a range from 929 to 4072 days. Based on univariate Cox regression analysis, 30 variables with P ≤ 0.25 were checked for collinearity, resulting in 9 variables eligible for inclusion in the Cox regression modelling: sex, height at shoulder (49.5 to < 53, 53 to 55.9 and 56.0 to 61.0 cm), coat colour, age at study start, BW at 8 and 10 years of age, change in LM from 8 to 10 years, LFR at 10 years and change in LFR from 8 to 10 years.

Backward elimination from a full model of nine variables resulted in a final model that included four variables: BW at 10 years of age, change in LM from 8 and 10 years, change in LFR from 8 and 10 years and age at study start (P ≤ 0.002, Table 3). There was an increased hazard of dying for every kg increase in BW at 10 years of age such that for each additional kg of BW at 10 years, dogs had a 19% higher hazard (HR = 1.19, P = 0.004). For both the change in LM and change in LFR variables, it was protective (lower hazard of dying) to have a higher LM and/or lower FM at 10 years of age compared to 8 years, although the HR for change in LM was very close to 1.0. For age at study start, older dogs had an increased hazard (HR = 1.69, P = 0.008).

**Table 3:** Results of univariable and multivariable Cox proportional hazards regression for 39 dogs

	Variable			
	BW at 10 years	Change in lean mass 8 to 10 years	Change in lean:fat 8 to 10 years	Age at start of study
Univariate analysis				
β (SE)	0.104 (0.045)	− 0.0004 (0.0002)	− 0.82 (0.48)	0.23 (0.20)
HR (SE)	1.11 (0.05)	0.9996 (0.0002)	0.44 (0.21)	1.25 (0.25)
HR 95% CI	1.017–1.210	0.9991–1.0001	0.17–1.13	0.85− 1.84
P-value	0.020	0.12	0.09	0.25
Multivariable analysis/adjusted model				
β (SE)	0.170 (0.059)	− 0.0012 (0.0003)	− 1.43 (0.45)	0.53 (0.20)
HR (SE)	1.19 (0.07)	0.9988 (0.0003)	0.24 (0.11)	1.69 (0.34)
HR 95% CI	1.06–1.33	0.9982, 0.9993	0.099–0.582	1.15–2.50
P-value	0.004	< 0.001	0.002	0.008

Body weight (BW), change in lean body mass and change in the ratio of lean to fat mass during middle age (~ 8 to 10 years of age) were all significant independent predictors of survival in the final regression model

**Fig. 5:**
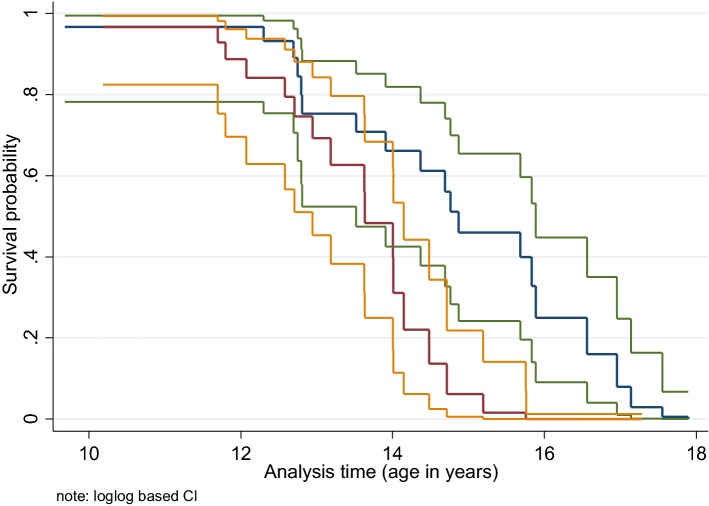
The survival function at mean values for covariates by body weight category at 10 years. The survival function with 95% confidence limits for the main Cox model (including the 4 variables: body weight at 10 years, change in lean mass from 8 to 10 years, change in lean to fat ratio from 8 to 10 years of age) and age at study start, evaluated at the mean value of the covariates and stratified by the mean body weight of 32 kg at 10 years of age. < 32 kg (blue with green CI) and ≥ 32 kg (brown with yellow CI)

**Fig. 6:**
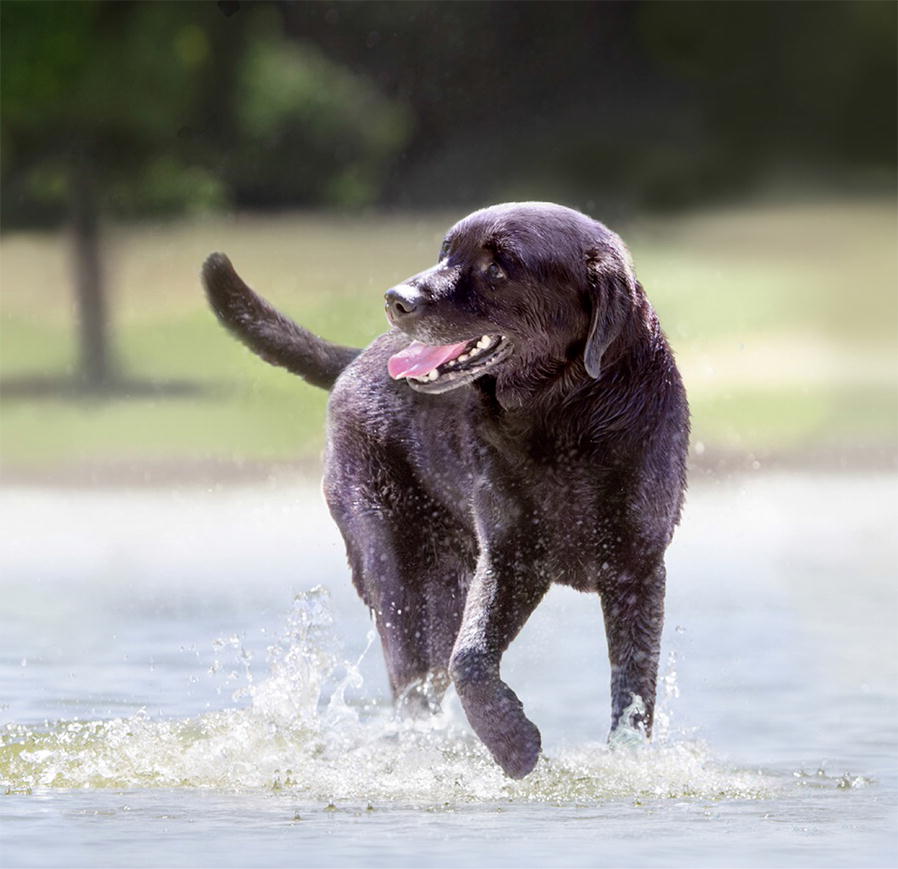
The longest living dog in the study pictured at 17 years of age illustrating the high level of healthspan accomplished by this dog. This was a male black Labrador called Utah, height at shoulders 55.9 cm, age at study start: 7.1 years, age at end-of-life 17.9 years. Body weight at 10 years = 28.1 kg, weight the month before end-of-life 28.8 kg, lean and fat body mass at 10 years: 21.4 and 5.8 kg, respectively (image courtesy of EUKANUBA^®^, UK)

The survival function of the final Cox model, evaluated at the mean value of the covariates, was plotted by stratifying the dogs based on the mean BW value of 32 kg at 10 years of age (Fig. 5). Four of five of the longest lived dogs (age at end-of-life > 16.6 years) had a BW < 32 kg at 10 years of age. Of the five dogs that died before 12.6 years of age (excluding two potential outliers that died at the youngest ages and earliest in the study), four had a BW ≥ 32 kg at 10 years of age. The longest living dog in the study, a male living to 17.9 years of age, was 7.1 years of age at the study start and at 10 years of age he had a BW of 28.1 kg, LM of 21.4 kg and FM of 5.8 kg, respectively, with a LFR of 3.7 (Fig. 6).

None of the two-way interactions were significant. The assumption of proportional hazard was not violated (P ≥ 0.2 for Schoenfeld residuals for each variable and a global P = 0.6; P = for the goodness-of-fit tests for five quantiles of risk). Two potential outlying observations were detected, and these were the two dogs that died at the youngest ages and earliest in the study (one of these dogs was also influential and one had very low influence). Rerunning the final model excluding these two dogs resulted in small changes (< 9%) for all point estimates of the HR. There were three additional dogs with the highest delta-beta residuals that could be considered to be influential. It appears that it was four rather heavy dogs that died fairly young that had highest influence; conversely, the other potential outlier that died at the youngest age had a BW of 25.5 kg at 10 years of age and showed very low influence. Removing the four dogs with the highest delta-betas resulted in similar small changes (< 7%) to the HR point estimates.

The sensitivity analyses yielded the same variables in the final model with approximately similar estimates of effect. Excluding the observations that were outside the ± 0.5 year range for ages 8 (n = 4) and 10 (n = 1) resulted in a small alteration in the HR for the change in LFR from 8 to 10 years of age. Adding BW at 12 years of age to the final model reduced the number of dogs included to 37 and changed the HRs for the other variables by < 15% and the variable itself was not significant (P = 0.6). Including average monthly BW as a time varying covariate did not indicate that monthly changes in BW influenced the final model (*p*_tvc_ = 0.14).

## Discussion

The present longitudinal study reports on BW and body composition factors related to age at end-of-life in a cohort of 39 adult Labrador retrievers. There are few studies that report on age related changes in body composition changes in humans, and even fewer longitudinal studies of people over 80 years of age, and factors that affect these changes such as physical activity. To our knowledge, no other study has reported similar data over adult life in dogs and adjusting for potential confounding factors. Investigating dogs of the same breed which received similar husbandry and feeding regimen consistently from adulthood to end-of-life poses a unique opportunity to study factors not related to variation in husbandry, exercise, health care, etc. which is inevitable when studying privately-owned dogs. Other studies that reported on longevity data from veterinary practices, insurance companies and owner surveys [5, 6, 20–22, 38] therefore provide less useful detailed comparisons as they will be inevitably biased by unmeasured factors related to diet, exercise, general husbandry and veterinary care as well as a lack of consistency of these factors over time. In fact, the suggested positive effects of consistent husbandry for the dogs in the present study is in line with findings from longitudinal human cohort studies reporting that healthy biological ageing was affected by lifetime protective factors including maintenance of normal weight across adult life, good prior health and positive health behaviours [39].

Early life environments including intrauterine, neonatal and early life have been reported as important for later life mortality [40] but it has also been suggested that there is no single factor or time point responsible for successful ageing but that ageing is due to lifelong accumulation of damage [41]. The ageing process leads to a decline in function of most organs and tissues, and includes sarcopenia, cognitive decline, diabetes, osteoarthritis and may be similar in dogs and humans, although the relative risk for individual diseases is not always shared [3]. Sarcopenia offers the potential for interventions to offset its commencement, progression or severity. Further, the co-existence of reduced muscle mass and increased fat mass, so called sarcobesity (sarcopenic obesity), has been acknowledged as an unfavourable relationship between lean and fat mass [29]. In humans the interest in sarcopenia lies with the amount of muscle mass and especially its strength [42, 43]. With muscle performance being clinically relevant for the frail and elderly there is less focus on the fat mass, aside from being cognisant of sarcopenic obesity. We therefore see reference to muscle mass, and particularly strength, rather than expression of the LFR in human studies (personal communication Dr. Emanuele Marzetti, Policlinico Universitario Agostino Gemelli, Italy, with DMM).

The current canine study does not address factors that may be important before ~ 6 years of age; however, it does show that, given that these dogs survived to adulthood, BW, LM, FM and LFR were important even in older age. This means that the status of a dog at a young age is not the only relevant time period for longevity as supported by the fact that BW at the study start alone was not statistically significant and thus not predictive of longevity. Instead, this study reports that body weight at late middle age (~ 10 ± 0.5 years of age) for these dogs was a predictor of longevity. An important advantage of this predictor is that it is easily evaluated in dogs hence supporting clinical relevance of the study finding, to maintaining and/or improving health of the ageing Labrador. Mean BW over time for the dogs in the present study was similar to that of the 24 ‘control’ dogs from the 1987 birth cohort who consumed more food than needed [13, 17, 33] and yet the dogs in the present study lived significantly longer [18]. This suggests that BW alone may not be a good predictor of survival/longevity; however, the earlier study did not report height at shoulder, making it difficult to evaluate the effect of the phenotype and size of the dogs. Future birth cohort studies that record potential confounders, including measures of physical activity, are needed to show whether early age status has a more profound effect on longevity than middle age status.

Few studies have investigated body composition and ageing in dogs and most have only looked at age at end-of-life and body composition measured on individual dogs at different ages [31, 32] and did not use repeated measures on dogs over time. Interestingly, the mean LM and change in LM between 9 and 13 years seen in the present study was similar to the ‘control’ fed (i.e. more generously fed) Labradors in the diet restriction study [13, 17, 33], albeit the dogs in the present study lived significantly longer. The dogs in the present study gained ~ 2 kg mean FM from 8 to 10 years of age whereas the ‘control’ dogs maintained fairly stable mean FM over these ages [13]. After age 11 years of age, our Labradors experienced an almost linear reduction in mean FM whereas the ‘control’ Labradors experienced an increase in FM of ~ 3 kg from 11 to 12 years of age (the last data point presented for this group) [13, 17]. The dogs in the present study had a relatively high mean PF throughout the study. For example, at 8 years of age the fat percent was 28% (for expected and exceptional dogs) and 30% (for long-lived dogs), respectively, and yet these dogs lived longer than the Labradors in the diet restriction study [17, 18]. This implies that relative fat mass may not be a crucial factor for longevity and raises the question of whether having a certain amount of FM in relationship to overall BW at a certain age is not strongly related to longevity. In fact, the variable fat percent at 10 years, with almost perfect correlation to lean mass percent at 10 years (data not shown), was offered to the full Cox regression model based on passing the criteria for univariate analysis and correlation analysis, but failed to remain in the model once other variables were accounted for. For FM, LM and fat percent over age, the pattern of fat mass change differs between our study and the diet-restriction diet. Further comparisons between the two studies are limited due to differences in recruitment age and study design. Another aspect of diet restriction is that it might reduce life quality if the dog is too hungry. To our knowledge there are no studies that have investigated stress, behaviour and life quality in hungry/calorie-restricted dogs. However, the genetic link between increased BW, obesity and increased appetite in Labrador and flat coat retrievers has been reported as strongly related to a deletion of the gene POMC via a change in the two peptides β-MSH and β-endorphin that are potent appetite regulators [44, 45]. The mode of action has been suggested as either through an additive [44] or recessive effect [45]. Knowledge of the genetic map of a Labrador retriever may aid in early prevention actions, in particular for dogs genetically prone to becoming overweight, and may also support breeding strategies to reduce the genetic predisposition for overweight and obesity in Labradors. Unfortunately, there was no genetic testing performed on the dogs in this study so the POMC status is unknown. It could be speculated that as all dogs in the present study were assessed daily by the welfare specialist to ensure life quality, it may be assumed that either none of the dogs had the POMC deletion or they were managed in a way that removed any negative effect of this deletion. As it has been suggested that dogs can influence the type and quantity of food offered to them by the owners and therefore access more food than optimal [46], the study design in the present study might have prevented an effect of a genetic mutation as any manipulation of the staff was likely low. Any future studies may establish the relationship between adequate BCS, hunger-management and life quality. The insights from the present study offer potential key factors to address to limit the growing problem of overweight and obese Labradors and promote expanded healthspan. The results also have potential cross-breed and cross-species applications.

The findings that both change in LM and change in LFR are related to survival have not previously been reported, although there are reports on one or the other factor having an impact [31, 32]; however, these earlier studies did not include dogs surviving to an exceptional age as reported here. The present study indicates that there are intricate relationships amongst the body composition variables and age, as shown by the final Cox model which included the change in LFR from 8 to 10 years of age, change in LM from 8 to 10 years, BW at 10 years and age at study start as factors associated with survival. Change in LM and FM with age were previously reported by the birth cohort group of 48 Labradors; estimating the mean LFR by age for the birth cohort dogs was performed using published mean age-specific values of lean and fat. The ‘restricted’ group had a LFR > 4.0 between 6 and 10 years of age and this was considerably higher than the dogs in the present study who had mean LFR < 3.0 which were more similar to the ‘control’ group; however, the dogs in the present study had a MST of 14.0 years that was significantly longer than the ‘control’ group which had a MST of 11.1 years. It is still not clear why the different cohorts of Labradors show such different phenotypes and lifespans. Indeed, none of the 48 birth cohort Labradors reached or exceeded an exceptional longevity of 15.6 years of age as compared to 28.2% of our present cohort of 39 dogs [18]. It may be argued that the ‘restricted’ group, with a mean FM of only about 3.5 kg (or ~ 16% body fat) at 6 years, and 4.5 kg (or ~ 19.5% body fat) at 8 and 10 years of age, with a MST of 12.9, may have been too thin. For comparison, our dogs classified as expected longevity had a median BCS of 3 (range 1–5; n = 9) at 8 years; for the exceptional group the median BCS at 10 years was also 3 (range 2–4.5; n = 11).

The decline in LM over time that commonly occurs with ageing calls for developing strategies to combat this loss. Through adopting for our dogs a life course perspective to healthy ageing [47] future research can look at early-life intervention strategies (“windows of opportunity”) that continue through all life-stages aimed at helping to offset or slow down sarcopenia developing. Ageing dogs are capable of metabolising dietary protein, providing us with simple feeding strategies that could help offset the development of sarcopenia. Whole-body protein turnover has been recorded in both young and ageing Beagles, average ages of 2 and 8 years respectively, fed isocaloric diets with 17.4, 25.8 and 34.0% protein on a DM basis over an 8-week feeding trial [48]. Rates of whole-body protein synthesis (WBPS) and whole-body protein degradation (WBPD) were positively correlated with dietary protein intake. Maintaining a “*high*” protein level (at least > 24% on an “as fed” basis or > 25% of calories) as provided to the dogs in the current study, would be practical for support of LM in healthy ageing dogs given that many other studies have found an age-related decline in endogenous protein synthesis [12, 49]. More recent data suggests that protein intake should be aimed at a minimum of 50% more than adult requirements, but further research is needed into what constitutes an *optimal* level in our healthy ageing dogs [50]. Additional reviews on protein and ageing have highlighted unequivocally that protein restriction is unnecessary in our healthy, older dogs [49].

As a result of the small sample of dogs with many observations recorded over ~ 10 years in the present study, some peculiarities emerged from the statistical analysis. For example, adding sex as a known confounder did not change the variables included in the final model, gave similar estimates of effect and sex was not significant. For simplicity, we therefore removed sex from the final model although it may be argued that a known confounding variable should be kept albeit non-significant. BW is easy to measure, although less clinically relevant to assess than lean and fat mass. As DEXA scans are not standard clinical equipment we need other reliable tools replacing DEXA scans in the clinic setting. Studies have reported on methods to estimate fat and/or muscle mass in dogs [51], for example, comparisons have been made between body fat estimations using DEXA, deuterium oxide, BCS (visual + palpation), and morphometric measurements in healthy dogs [52]. This initial study on 23 dogs showed an overall high correlation between DEXA scan, BCS and morphometric measurements, but as within dog estimates varied quite significantly (> 5 to 10% difference), it would be advisable to repeat this study in more dogs of various types and breeds to establish the accuracy of the BCS and morphometric measurements. Regular veterinary assessment of *both* the BCS and muscle condition score (MCS) should be undertaken to monitor overall body composition, namely adipose tissue, but also muscle mass. Relying on just taking body weight measurement could potentially mislead the clinician to erroneous conclusions, especially if there is sarcopenic obesity with a parallel *increase* in body weight, thus rendering this measure as rather insensitive [27]. In fact, the World Small Animal Veterinary Association (WSAVA) have developed a practical nutritional assessment toolkit that can help evaluate each patient for both BCS and MCS [53], and other comprehensive reviews of ageing dogs are available [49].

Previous studies have reported on the predictive ability of the body composition variables from DEXA scans performed at the time of end-of-life and at 1 and 2 years before end-of-life. It was not possible to replicate this type of analysis in the present study as DEXA scans were not performed at the same ages for all the dogs. However, in the present study the dogs were recruited in middle age and then initially acclimatised to reduce their BW before starting the study so as to reach an optimal target BCS of 3 on a 5-point scale. This BW reduction was required for the majority of dogs before entering the study. The increased healthspan and longevity reported for these dogs indicates that rather late efforts to control BW and FM is likely to have a positive effect on the dog’s longevity. This should be encouraging to owners, as well as their veterinarian, when implementing weight reduction strategies having realized at a rather late stage of adulthood that their dog is too heavy for their size.

For privately owned dogs, the decision of euthanasia is often based on a combination of factors relating to the dog, the owner(s) and the veterinarian [54]. In the present study the decision was made by a team that evaluated the dogs’ welfare and quality of life, hence removing some of the variation in the decision making process; we suggest that this was likely to result in a more accurate age of end-of-life that reflected the age when the dog’s life needed to end based only on health and welfare.

The dogs in the present study received a balanced commercial ‘senior’ diet. Studies are being performed to identify modulators that can be added to a diet to prolong life. For example, recent studies have investigated the possibility of extending life span though the immune modulator rapamycin [3], an inhibitor of the mammalian target of rapamycin (mTOR) signalling pathway and which has demonstrated an effective anti-ageing effect in various model organisms (yeast, worms, flies, mice) [55–58]. The mTOR signalling pathway serves as a central regulator of cell metabolism, growth, proliferation and survival [59], it is also inhibited by calorie restriction [60]. Another candidate for clinical testing is metformin, a biguanide antihyperglycaemic agent [3]. The role of the gut microbiota is also being investigated as a potential strategy for longevity extension through exploitation of the bidirectional communication system of the gut-brain-axis (GBA) [61], specially targeting the effect of age-related chronic inflammatory processes, inflammageing (as previously mentioned), for example to reduce its impact on muscle ageing, the focus being firmly aimed at sarcopenia [62].

A recent publication that also evaluated data from this present cohort of 39 Labradors, together with a second cohort of 41 Labradors, reported finding a profile of changes indicative of inflammageing [9]. While this study did not explore any association of inflammation and body composition measures in these ageing dogs it does help highlight those pathways that may be valuable for developing preventive and therapeutic interventions against muscle ageing in dogs. Although these prospects are promising, the present study indicates that consistency in feeding, activity and exercise and health care were key to a long healthspan in these dogs.

One potential limitation of this study is that the cohort consisted of dogs that were recruited, at the start of the study in July 2004, at a median age of 6.5 years (range of ages from 5.4 to 8.5 years), and as such constitute a sample of the Labrador population that had survived to adulthood. As the dogs had to survive and be healthy at recruitment, some caution must be applied when extrapolating to survival from birth. However, this is somewhat contradicted by the findings in the study: the age of the dog at the start of study was negatively associated with age at end-of-life: The study suggests that there was not a linear survivorship effect by enrolling dogs aged > 5 years; in fact, the final model suggests that the older the dog at enrolment, the greater the hazard of end-of-life. It may be that there is a threshold for longevity so that if the dog survives to a certain age it is likely to live longer. This thought is in line with the concept of population heterogeneity proposing that if there is variation in the underlying frailty within a population (i.e., demographic heterogeneity) at the individual-level, at each age individuals with higher frailty to a specific factor are more likely to die. Hence, the impact/importance of this factor may decrease over time as more resilient individuals are left. This differential selection can cause different mortality patterns for the population as a whole compared to individuals [63]. Ideally, individuals should be randomly selected to studies to account for the individual-level heterogeneity [64]. In this study the life-style and environmental factors were kept similar to all individuals, which may reduce their influence on the population longevity.

Another aspect to consider is that 32 of the dogs were recruited from the same breeder and many were littermates. The Cox regression model took the clustering of dogs within litters into account. Adding breeder to the final model as a fixed effect was non-significant (P = 0.83, data not shown). Running the final model separately for dogs from the same breeder (n = 32) gave similar results as when including all dogs, as expected by the large contribution of dogs. However, the point estimate HR for age at start of study was lower albeit no longer statistically significant. On the other hand, when running the model for the dogs from other breeders (n = 7), the HR for age at start of study was larger (3.8) as was HR for BW at 10 years (1.29) whereas the HR for change in LFR was somewhat smaller (0.02; data not shown) but explanatory variables were non-significant due to the low number of dogs. This suggests that the breeder contributing with many dogs had its largest impact on longevity by potentially reducing the effect of age at recruitment and BW at 10 years.

## Conclusions

These results suggest that for this cohort of Labrador retrievers even rather late-life control efforts, after 8 years of age, on body weight and the relationship between body lean and fat mass, influenced survival. Such “windows of opportunity” can be used to develop healthcare strategies that would help promote an increased healthspan in dogs.

## Data Availability

The datasets generated and/or analysed during the current study are not publicly available due to being held by two independent commercial companies but are available from the corresponding author on request.

## Abbreviations

BCS: body condition score
BW: body weight
CI: confidence interval
DEXA: dual-energy x-ray absorptiometer
FM: fat mass
HR: hazard ratio
LFR: lean to fat ratio
LM: lean mass
MST: median survival time
PF: percent fat mass
PL: percent lean mass
SD: standard deviation

## Footnotes

1 This is correction to what was reported in the earlier paper Adams et al. [19] of 33 dogs.

2 At the time of the study’s commencement in July 2004 the brand EUKANUBA^®^ was owned by Procter and Gamble, Cincinnati, OH, USA. On January 1st 2015 the brand was purchased for Europe only by Spectrum Brands, Middleton, WI, USA and continues to be sold under the name EUKANUBA^®^ in Europe.

3 Assessments were conducted by animal welfare specialists, animal husbandry staff and healthcare staff (attending veterinarian, veterinary nurses) to assess quality of life using a 10-point Likert scale ratings to assess food/water intake (hunger/thirst), pain/discomfort, mobility, hygiene, happiness and number of “good days”. When the dog was having more “bad days” than “good days” then the attending veterinarian convened a panel to discuss an end-of-life decision; original concept, Oncology Outlook, by Dr. Alice Villalobos, Quality of Life Scale Helps Make Final Call, VPN, 09/2004; scale format created for author’s book, Canine and Feline Geriatric Oncology: Honouring the Human-Animal Bond, Blackwell Publishing, 2007. Revised for the International Veterinary Association of Pain Management (IVAPM) 2011 Palliative Care and Hospice Guidelines.
